# Identification of variants in SWI/SNF complex genes associated with neurodevelopmental disorders

**DOI:** 10.3389/fgene.2025.1511796

**Published:** 2025-07-08

**Authors:** Chen Liang, Haihong Shi, Yanjuan Chen, Xia Wang, Jieyuan Jin, Liqun Su, Lijun Tang, Huihong Li, Fei Ling, Haoxian Li, Yanghui Zhang

**Affiliations:** ^1^ Department of Clinical Laboratory, Jiangmen Maternal and Child Health Care Hospital, Jiangmen, China; ^2^ Center for Medical Genetics, Jiangmen Maternal and Child Health Care Hospital, Jiangmen, China; ^3^ Children Rehabilitation Center, Jiangmen Maternal and Child Health Care Hospital, Jiangmen, China; ^4^ Department of Pediatrics, Xiangya Hospital, Central South University, Changsha, China; ^5^ School of Medicine, Shaoxing University, Shaoxing, China; ^6^ Ultrasonography Department, Jiangmen Maternal and Child Health Care Hospital, Jiangmen, China; ^7^ Nursing Department, Jiangmen Maternal and Child Health Care Hospital, Jiangmen, China; ^8^ Reproductive Medicine Center, Jiangmen Maternal and Child Health Care Hospital, Jiangmen, China

**Keywords:** ARID2, ARID1B, SMARCC2, SWI/SNF complex, Coffin-Siris syndrome, neurodevelopmental disorders

## Abstract

**Introduction:**

Neurodevelopmental disorder (NDDs) such as intellectual disability, developmental delay encompasses a diverse group of conditions caused by the disruptions in the central nervous system (CNS) during development. Variants in the SWItch/Sucrose non-fermentable (SWI/SNF) complex genes are significant contributors to NDDs. ARID2, ARID1B, and SMARCC2 are important subunits of the SWI/SNF complex, and their variants can also result in Coffin-Siris syndrome (CSS), a type of NDDs characterized by CNS disorders, global developmental delay, visual/hearing impairment, distinct facial features, and congenital heart disease (CHD).

**Methods:**

Three NDDs families were recruited, and whole-exome sequencing and Sanger sequencing were used to detected their causative variant.

**Results:**

We described their symptoms and identified three variants of SWI/SNF complex genes unreported in disease cohorts, including a deletion variant of ARID2 (NM_152641: c.2901delC, p.Asn967LysfsX2), an insertion variant of ARID1B (NM_001374828: c.6532_6533insT, p.Trp2178LeufsX34), and a missense variant of SMARCC2 (NM_003075: c.2920C>G, p.Pro974Ala). Additionally, we compiled known variants in *ARID2*, *ARID1B*, and *SMARCC2* associated with CSS/NDDs.

**Conclusion:**

We reported three SWI/SNF variants in three NDDs families. Our identification broadened the variant spectrum of SWI/SNF genes and contributed to the genetic counseling and molecular diagnosis of NDDs.

## 1 Introduction

Neurodevelopmental disorders (NDDs) represent a diverse group of conditions characterized by disruptions in the central nervous system (CNS) during development. They are among the most prevalent diseases in childhood and adolescence, with an estimated incidence of 17% among children aged 3 to 17 in United States (Zablotsky et al., 2019; Penttila et al., 2024). NDDs include intellectual disability, attention deficit hyperactivity disorder (ADHD), autism spectrum disorders (ASD) and specific learning, motor, and communication disorders (Zablotsky et al., 2019; Valencia et al., 2023; You et al., 2024). Moreover, NDDs frequently co-occur with other anomalies, such as facial dysmorphisms, congenital heart disease (CHD), and growth retardation, seriously affecting the patients’ health and quality of life (Billotte et al., 2021; Tolezano et al., 2024).

Genetic etiologies are responsible for NDDs, and variants in the SWItch/Sucrose non-fermentable (SWI/SNF) complex genes have been identified as critical contributors (Valencia et al., 2023). The SWI/SNF complex is also known as the BRG1/BRM-associated factor (BAF) complex in mammals and plays an essential role in ATP-dependent chromatin remodeling to determine gene accessibility and expression (Ahmad et al., 2024). Approximately 30 proteins have been identified as SWI/SNF complex subunits, of which, ARID2, ARID1B, and SMARCC2 are core components (Agaimy and Foulkes, 2018). ARID2 is required for stability of the SWI/SNF complex, and ARID1B and SMARCC2 exhibit key enzymatic activities (Kadam et al., 2000; Wang et al., 2004; Kadoch and Crabtree, 2015). Their variants are associated with NDDs, cancer, and Coffin-Siris syndrome (CSS). CSS is characterized by a constellation of NDDs, global developmental delay, visual/hearing impairments, distinct facial features, and CHD (Wang et al., 2004). And cancer-associated variants are often somatic variants and lead to subunit deletions or gene silencing (Wang et al., 2021).

In this study, we reported three subjects with NDDs and additional symptoms and identified three variants in SWI/SNF complex genes: a deletion variant of *ARID2* (NM_152641: c.2901delC, p.Asn967LysfsX2), an insertion variant of *ARID1B* (NM_001374828: c.6532_6533insT, p.Trp2178LeufsX34), and a missense variant of *SMARCC2* (NM_003075: c.2920C>G, p.Pro974Ala), which were not identified in affected individuals. Our identification extended the variant spectrum of SWI/SNF genes. We also compiled known variants associated with NDDs/CSS in *ARID2*, *ARID1B*, and *SMARCC2*, contributing to the genetic counseling and molecular diagnostics in NDDs/CSS.

## 2 Materials and methods

### 2.1 Subjects

This research received approval from the Review Board of Jiangmen Maternal and Child Health Care Hospital (No. 112[2022], Data: 2022.11). We recruited three families affected by NDDs with accompanying systemic disabilities (Family 1–3). The guardians of probands provided written informed consent for their children’s participation in this study and for the publication of related information.

### 2.2 Karyotype analysis and chromosomal microarray analysis

Cells of the Proband 1 was acquired by amniocentesis. Cells was stained with Geimsa for 15 min, and then washed and dried. Karyotype analysis was performed using a light microscope.

Chromosomal microarray analysis was conducted in the Proband 1 and 3 using the Affymetrix Cytoscan 750K chip (Affymetrix), performed by Genergy Bio-technology (Shanghai, China).

### 2.3 Whole-exome sequencing and sanger sequencing

Genomic DNA was extracted from the peripheral blood of participants and sent to Berry Genomics Company Limited (Beijing, China) for whole-exome sequencing (WES) following protocols as our previously described (Liang et al., 2023). Based on data from GnomAD database (http://gnomad.broadinstitule.org), rare variants (detection rate <0.001) within exons and/or splicing sites were retained for further analysis. Variants predicted to be benign or likely polymorphic (score ≤10 in CADD) were excluded, using tools such as MutationTaster (http://www.mutationtaster.org), SIFT (http://provean.jcvi.org/index.php), and CADD (https://cadd.gs.washington.edu/snv). OMIM database (https://www.omim.org) provided annotations of phenotypes and inheritance patterns of the variant genes. MUpro (https://ics.uci.edu/∼baldig/mutation.html) was used to predict the protein stability changes for single-site variants. Pathogenicity classification of the variants adhered to the standards and guidelines set by the American College of Medical Genetics and Genomics (ACMG) (Richards et al., 2015).

The variants were verified by Sanger sequencing. Variant sites and their flanking sequences were acquired from the NCBI database (https://www.ncbi.nlm.nih.gov/gene). The following primer pairs were designed for sequencing: *ARID2* f: 5′-CCA​ACA​AAG​CGT​AGT​GAT​TGT​AAG-3′ and r: 5′-GGT​GAA​TGT​TGC​TGC​TGT​TG-3’; *ARID1B* f: 5′-GAA​AGA​GGA​GGA​TGA​GGA​CAA​G-3′, and r: 5′-CTG​ACG​ACT​AAA​TGG​AGG​AGT​G-3’; *SMARCC2* f: 5′-GAC​AGA​CAA​GCC​TTC​CAC​AT-3′, and r: 5′-GAC​AGA​CAA​GCC​TTC​CAC​AT-3’.

### 2.4 Three-dimensional protein modeling

Three-dimensional wild-type protein models of ARID2 (Q68CP9), ARID1B (Q8NFD5), and SMARCC2 (Q8TAQ2) were obtained from the AlphaFold database (https://alphafold.ebi.ac.uk). Mutant protein models were subsequently generated using PyMol: 1) using the “Wizard-Mutagensis-Protein” tool to replace wild-type amino acids by mutant amino acids; 2) different domains annotated with different colors (domain data from Uniprot [https://www.uniprot.org/]; 3) deleting the missing regions of the mutant proteins of ARID2 and ARID1B; 4 showing the mutant and related amino acids in SMARCC2 with stick modeling).

## 3 Results

### 3.1 Clinical description

**FIGURE 1:**
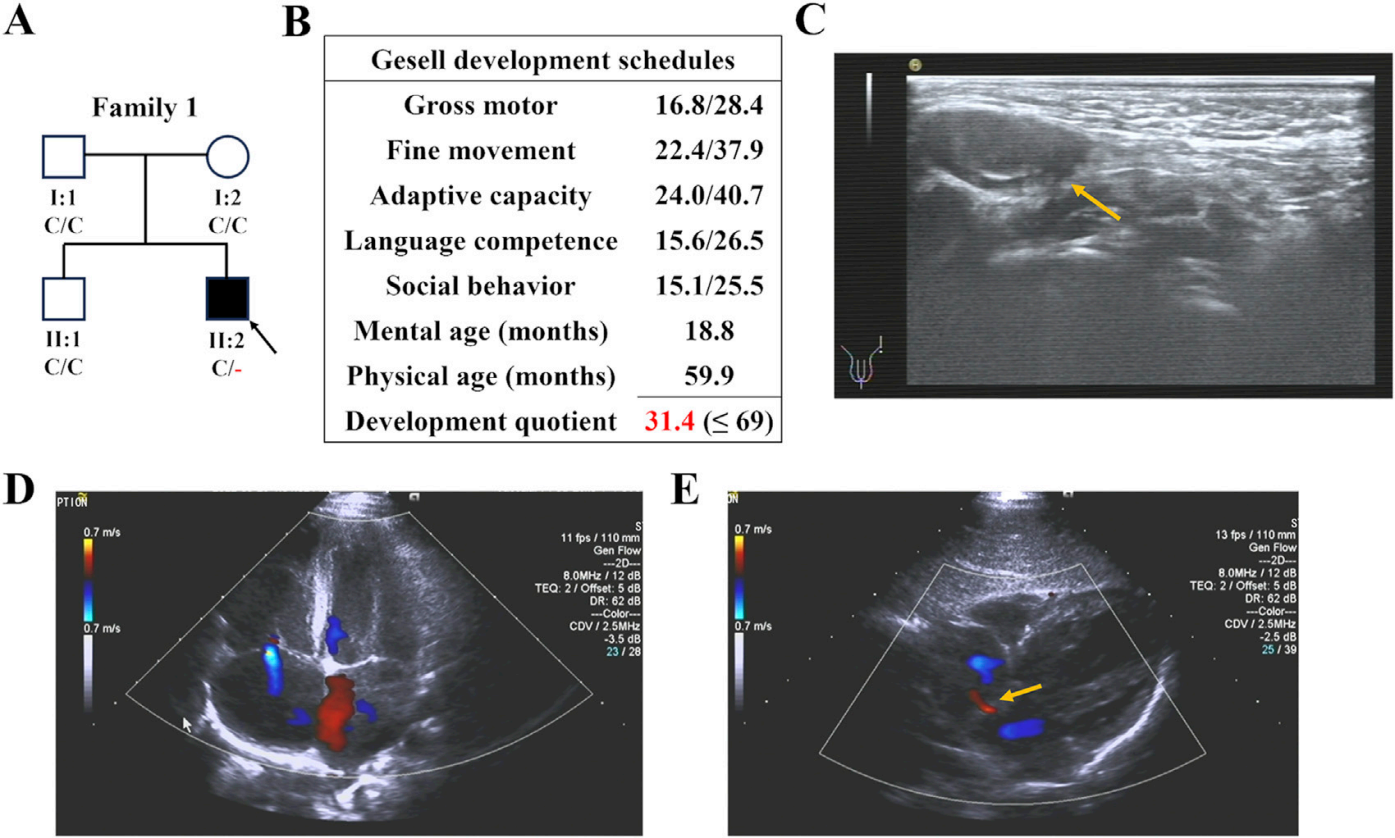
The family pedigree and clinical details of Proband 1. **(A)** The family pedigrees of Proband 1. The black symbols represent the affected members, arrows indicate probands, and red fonts represent variants in this study. **(B)** The result of Gesell development schedules of Proband 1, showing his developmental delay. **(C)** Ultrasonic testing showed that Proband 1 had left cryptorchidism. **(D,E)** Cardiac color ultrasound showed that Proband 1 had tricuspid and pulmonary regurgitation **(D)** and patent foramen ovale **(E)**.

Proband 1 (II:2) was a five-year-old boy from Family 1 (Figure 1A). He presented to our hospital with NDDs, speech delay (unable to speak), visual impairment, and hypokinesia (ambulation with help and unable to jump). Gesell development schedule test is a classical assessment mothed of early child development (Yang et al., 2020). Gesell development schedule assessments revealed a development quotient of 31.4 (≤69), indicating significant developmental delay (Figure 1B). Through further medical evaluations and inquiries, we found that the proband had an adenoid face, left cryptorchidism (Figure 1C), tricuspid and pulmonary regurgitation with reflux areas of 0.6 and 0.7 cm^2^ respectively (Figure 1D), patent foramen ovale (PFO) with an aperture of 1.2 mm (Figure 1E), feeding difficulties, and a history of recurrent infection (Table 1). His parents (I:1 and I:2) and sibling (II:1) were unaffected.

**TABLE 1:** The clinical and genetic details of NDDs subjects in this study.

Proband	Age (years)	Gender	Symptoms	Variant	Pathogenicity prediction	GnomAD v4.1.0	ACMG classification
1	5	M	Developmental delay, speech delay, tricuspid regurgitation, pulmonary regurgitation, patent foramen ovale, adenoid face, cryptorchidism, feeding difficulties, visual impairment, attention deficit hyperactivity disorder, repeated infection	*ARID2*: NM_152641: c.2901delC, p.Asn967LysfsX2	MutationTaster: D SIFT: CADD: 18.4	-	Pathogenic (PVS1, PS2, PM2)
2	2	F	Global developmental delay, hearing loss, obstructive sleep apnea syndrome	*ARID1B*: NM_001374828: c.6532_6533insT, p.Trp2178LeufsX34	MutationTaster: D SIFT: CADD: 32.0	-	Pathogenic (PVS1, PS2, PM2)
3	4	M	Intellectual disability, atrioventricular septal defect	*SMARCC2*: NM_003075: c.2920C>G, p.Pro974Ala	MutationTaster: D SIFT: D CADD: 14.8	0.00001	Likely pathogenic (PS2, PM1, PP3)

F, female; M, male; D, disease-causing; -, nonexistence data; ACMG, american college of medical genetics.

**FIGURE 2:**
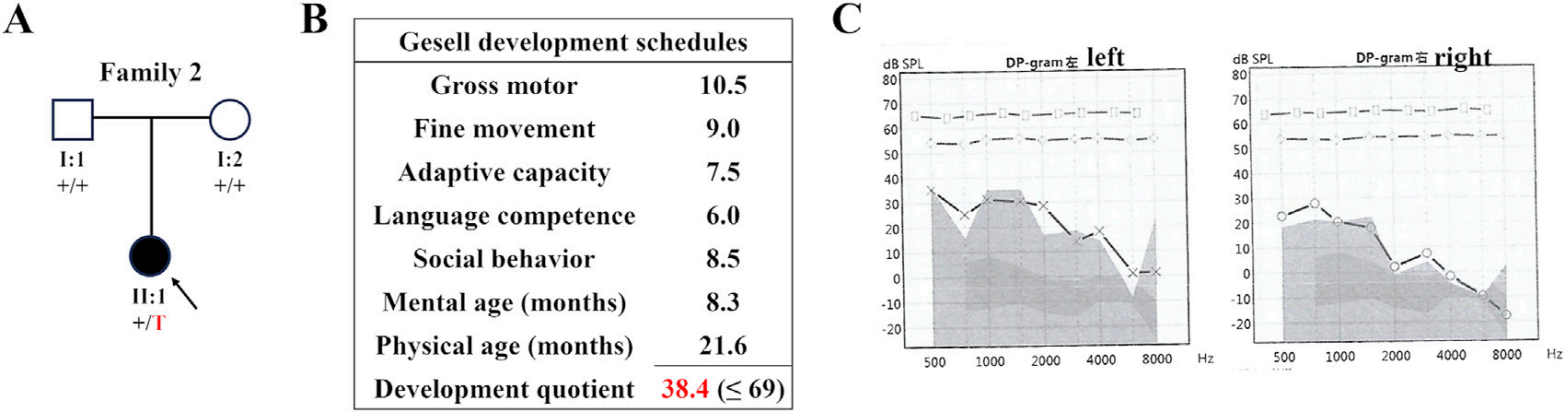
The family pedigree and clinical details of Proband 2. **(A)** The family pedigree of Proband 2. The black symbols represent the affected members, arrows indicate probands, and red fonts represent variants in this study. **(B)** The result of Gesell development schedules of Proband 2, showing her developmental delay. **(C)** The audiogram of Proband 2 showing her hearing loss.

Proband 2, a two-year-old girl (Figure 2A), exhibited global developmental delay (growth retardation, speech and motor delay; Figure 2B), binaural hearing loss (Figure 2C), and obstructive sleep apnea syndrome (OSAS). Proband 3, a four-year-old boy, was diagnosed with intellectual disability and atrioventricular septal defect (AVSD; Table 1). Similar to the first case, their parents did not present any of these conditions.

### 3.2 Genetic analysis

**FIGURE 3:**
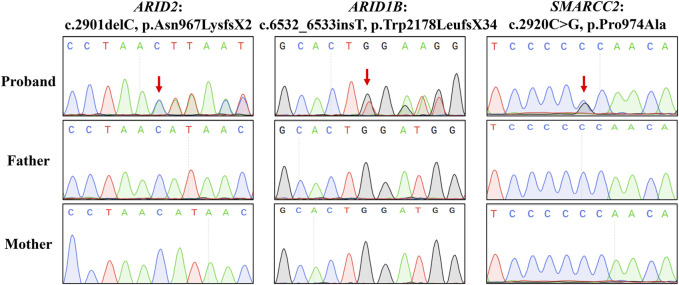
Sanger sequencing results. Sequence chromatograms are left to right the *ARID2* variant of Proband 1, the *ARID1B* variant of Proband 2, and the *SMARCC2* variant of Proband 3.

WES was employed to identify three variants in SWI/SNF genes in these three families: a novel deletion variant of *ARID2* (NM_152641: c.2901delC, p.Asn967LysfsX2) in Proband 1, a novel insertion variant of *ARID1B* (NM_001374828: c.6532_6533insT, p.Trp2178LeufsX34) in Proband 2, and a known missense variant (recorded by GnomAD database) of *SMARCC2* (NM_003075: c.2920C>G, p.Pro974Ala) in Proband 3 (Table 1). Sanger sequencing confirmed that these variants were all *de novo* (Figure 3). In addition, we did not find potential causative CNVs in them by karyotype analysis, microarray analysis or WES analysis.

**FIGURE 4:**
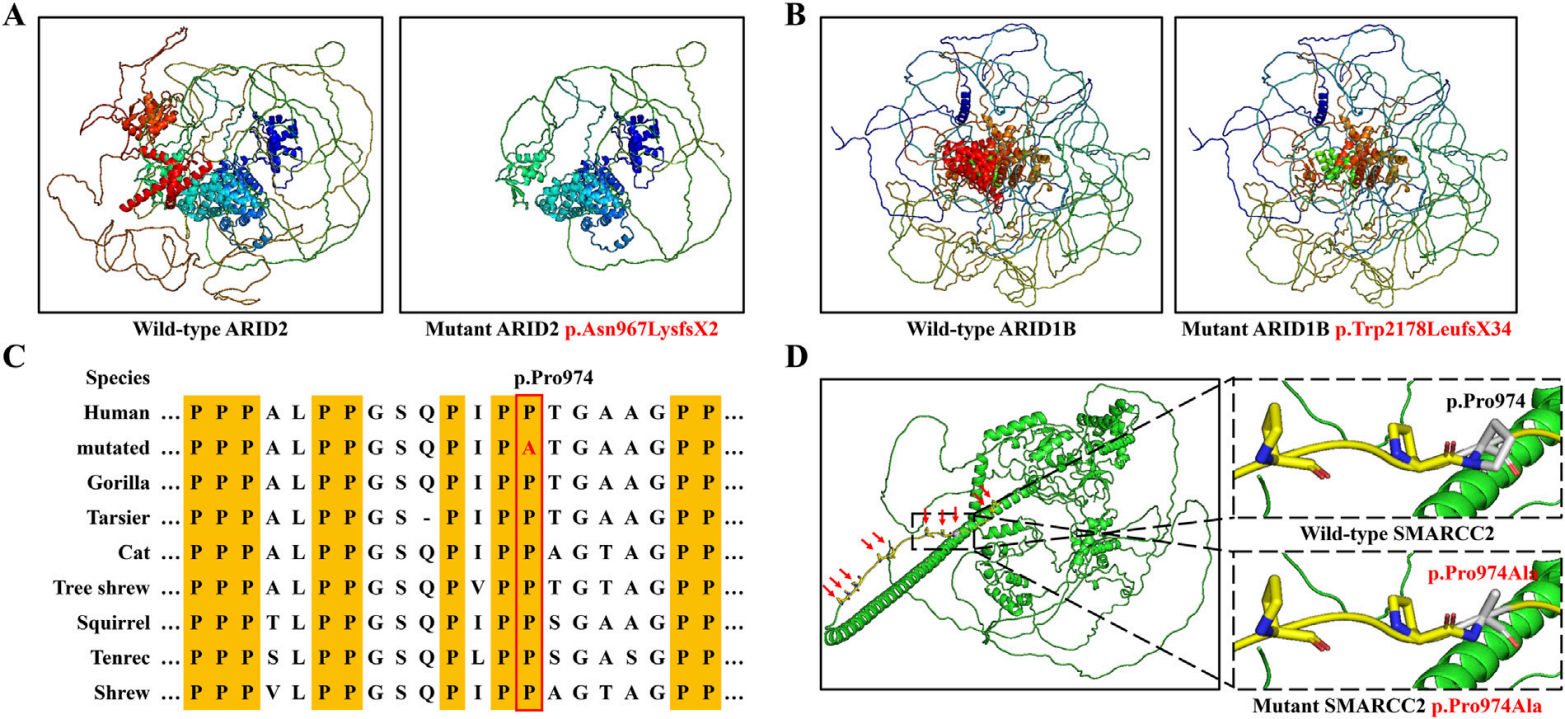
Three-dimension modeling of mutant proteins and the analysis of amino acid conservation. **(A)** Protein models of ARID2 with wild type and the variant p.Asn967LysfsX2. **(B)** Protein models of ARID1B with wild type and the variant p.Trp2178LeufsX34. **(C)** SMARCC2 peptide sequences surrounding the mutated residues with multiple interspecies alignments. **(D)** Protein models of SMARCC2 with wild type and the variant p.Pro974Ala.

Three-dimensional modeling showed that variants p.Asn967LysfsX2 in ARID2 and p.Trp2178LeufsX34 in ARID1B both produced truncated proteins lacking functional domains typically composed of α-helixes (Figures 4A,B). In adherence to the ACMG guidelines, both of them were classified as “Pathogenic” for following reasons (Table 1): (1) They were frameshift variants in genes where loss of function (LOF) is a known disease mechanism (PVS1). (2) They were *de novo* variants (PS2). (3) They were absent in GnomAD (v4.1.0) database, which was the world’s largest databases of human genome variation (PM2). In addition, MutationTaster also predicted these variants as disease-causing.

The SMARCC2 variant p.Pro974Ala occurred within an intrinsically disordered protein region (IDR) rich in proline residues, and p.Pro974 was highly conserved across evolution (Figures 4C,D). MUpro predicted that this variant would decrease the stability of SMARCC2 (ΔΔG = −0.3614). This variant was considered “Likely pathogenic” based on ACMG criteria: (1) it was a *de novo* variant (PS2); (2) it was located in the core region of IDRs (PM1); (3) it was predicted to be disease-causing by bioinformatics analysis (PP3; Table 1). Consequently, we reasoned that these three variants were genetic etiologies in these families.

## 4 Discussion

ARID2, ARID1B, and SMARCC2, as SWI/SNF members, possess helicase and ATPase activities crucial for the transcriptional activation and repression of certain genes through chromatin remodeling to alter DNA-nucleosome topologies (Bogershausen and Wollnik, 2018). ARID2 and ARID1B belong to AT-rich DNA interacting domain-containing (ARID) family proteins and play significant roles in embryonic patterning, cell lineage gene regulation (especially in neural progenitors and neurons), cell cycle control, and chromatin modification (Wang et al., 2004). SMARCC2 regulates embryogenesis and cortical neurogenesis and determines cortical size and thickness (Tuoc et al., 2013). Deletion of SMARCC2 has been shown to result in learning and behavioral adaptation deficiencies in mice. In human, defects of these three genes have been implicated in NDDs, with most variants arising *de novo* (Valencia et al., 2023). In this study, we identified three variants in *ARID2* (c.2901delC, p.Asn967LysfsX2), *ARID1B* (c.6532_6533insT, p.Trp2178LeufsX34), and *SMARCC2* (c.2920C>G, p.Pro974Ala) in participants with NDDs, and these variants were *de novo*. The *ARID2* and *ARID1B* variants were frameshift variants producing premature stop codons, which likely caused either truncated proteins or nonsense-mediated mRNA degradation, disrupting protein functions and being responsible for NDDs in these patients. The *SMARCC2* variant occurred within an IDR, where proline residues are densely distributed. Given the critical role of liquid-liquid phase separation (LLPS) in transcriptional regulation and its reliance on IDRs, the substitution of proline with alanine within the IDR may impair the transcriptional regulatory function of SMARCC2, warranting further investigation (Tsang et al., 2020).

*ARID2*, *ARID1B*, and *SMARCC2* are classical CSS genes, with *ARID1B* being the most common (Schrier Vergano et al., 1993). Their variants are linked with CSS6 (OMIM 617808), CSS1 (OMIM 135900), and CSS8 (OMIM 618362) respectively (Bogershausen and Wollnik, 2018). NDDs, for instance intellectual disability, may be the most core phenotypes of CSS. Other CSS symptoms include facial features, sparse hair, hypoplastic nails, and short stature (Schrier Vergano et al., 1993). In this study, we diagnosed Proband 1 with CSS6 based on his common CSS phenotypes and *de novo ARID2* variant. Notably, he presented with rare phenotypes, such as cardiac abnormalities and feeding difficulties. Cryptorchidism and repeated infection may be potential phenotypes for CSS6 that had not been reported (Xia et al., 2023). Our report enriched phenotype profile of CSS6. However, despite other two subjects also harbored variants in CSS genes and presented NDDs, the lack of comprehensive clinical details precluded the definitive CSS diagnosis.

**FIGURE 5:**
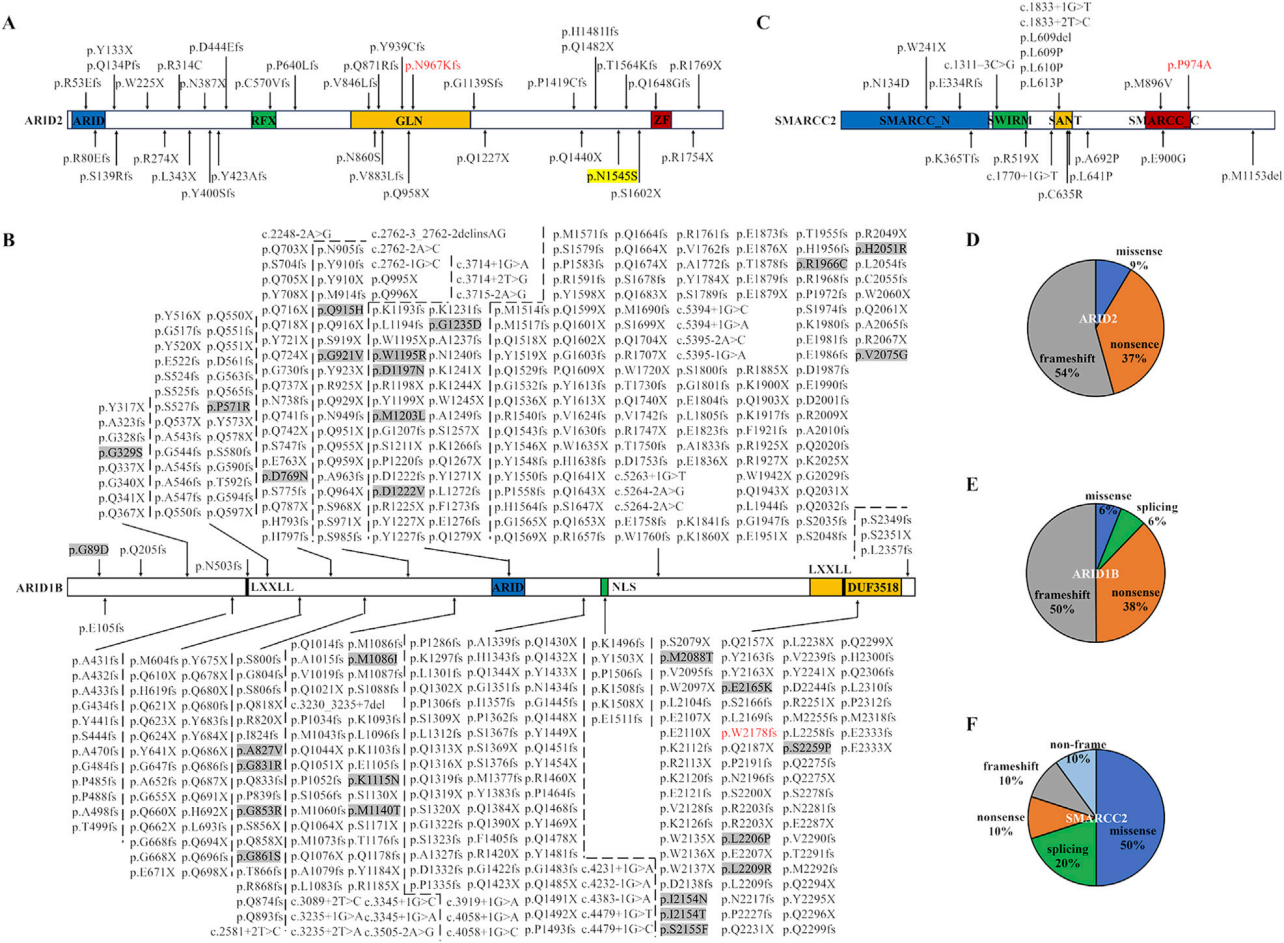
Pathogenic variants in *ARID2*, *ARID1B*, and *SMARCC2* were reported in databases, literature, and this report. **(A–C)** Protein structure domains and variant collection of ARID2 **(A)**, ARID1B **(B)**, and SMARCC2 **(C)**. The red fonts represent our variants. ARID, AT-rich DNA interacting domain; RFX, regulatory factor-like DNA binding domain; GLN, glutamine enriched area; ZF, C2H2 zinc-finger structures; LXXLL, a motif with a special amino acids sequence; NLS, nuclear localization signal. The protein-protein interaction modules maned DUF3518 domain, SWIRM domain, and SANT domain. Missense variants in *ARID1B* are highlighted by grey backgrounds. **(D–F)** The proportion of variant types in *ARID2* **(D)**, *ARID1B* **(E)** and *SMARCC2* **(F)**.

At least 35 *ARID2* variants, 483 *ARID1B* variants, and 20 *SMARCC2* variants had been reported in CSS and NDDs (Figures 5A–C; data from HGMD database [https://www.hgmd.cf.ac.uk/ac/search.php], ClinVar database [https://www.ncbi.nlm.nih.gov/clinvar/], and literature) (Sim et al., 2015; Machol et al., 2019; van der Sluijs et al., 2019; Yi et al., 2022; Xia et al., 2023; Schmetz et al., 2024; Schrier Vergano, 2024). ARID2 includes an ARID domain, a regulatory factor-like DNA binding domain (RFX), a glutamine enriched area (GLN), and a C2H2 zinc-finger structures (ZF) (Xia et al., 2023). These variants are scattered in ARID2 without preferences or aggregation. Analogously, 91% *ARID2* variants are frameshift or nonsense variants, which damage the functions of not only the certain domain but also the whole protein (Figure 5D). Our *ARID2* variant was frame shift occurring in the GLN domain.

ARID1B includes an ARID domain, a nuclear localization signal (NLS), a DUF3518 domain, and two LXXLL motifs (Figure 4B). Our *ARID1B* variant was positioned in the DUF3518 domain which can interact with the helicase subunits in BAF complexes (Sim et al., 2015). Like *ARID2* variants, 94% *ARID1B* variants are null variants divided into 50% frameshift variants, 38% nonsense variants, and 6% splicing variants (Figure 5E). Our variant also a frameshift variant. Only 29 variants (6%) are missense variants, and half of them occurred in the ARID domain and DUF3518 domain, suggesting the importance of these domains for ARID1B functions.

SMARCC2 includes an SMARCC_N-terminal domain, an SWIRM domain (predicted to mediate specific protein-protein interactions in the assembly of chromatin-protein complexes), a protein-protein interaction module maned SANT domain, and an SMARCC_C-terminal domain (Figure 5C) (Aravind and Iyer, 2002; Machol et al., 2019). Half of *SMARCC2* variants are missense variants, mainly distributed in the SANT domain, followed by the SMARCC_C domain (Figure 5F). Our missense variant happened in the SMARCC_C domain, and other two SMARCC_C domain variants (p.Met896Val and p.Glu900Gly) had been also identified in patients with NNDs (Machol et al., 2019). These findings indicated the relevance of this domain to NDDs. In addition, studies of the SMARCC_C domain were lacking, and investigations of impacts induced by related variants were restricted to medical genetics. Thus, their pathogenic mechanism was still unknown. Given that the domain covers a long IDR, the protein interactions and LLPS through the IDR may be a potential research idea.

Excepted for CSS, the SWI/SNF complex defect can also lead to cancers, especially those variants producing truncated proteins (Mittal and Roberts, 2020). For instance, germline missense variants in *SMARCA4* cause CSS, while germline null variants are responsible for cancers (Bogershausen and Wollnik, 2018). In *ARID2* and *ARID1B*, most cancer-associated variants are somatic and null variants, and only few germline variants or copy number variations are reported to be associated with cancers, such as the *ARID2* variant c.4634A>G, p.Asn1545Ser identified in an acute lymphoblastic leukaemia case and the 6q25 deletion (including *ARID1B*) in papillary thyroid cancer (Sausen et al., 2013; Vengoechea et al., 2014; de Smith et al., 2019; Wang et al., 2021). Van der Sluijs et al. (2019) reported 143 CSS/NDDs patients with *ARID1B* variants and only found one boy with malignancy (van der Sluijs et al., 2019). In our compilation and report of *ARID2* and *ARID1B* variants, all patients with truncated variants did not have cancers. It suggested that pathogenic germline variants in *ARID2* and *ARID1B* did not increase the cancer risk, which should be verified by more investigations.

## 5 Conclusion

In this study, we identified three SWI/SNF variants in NDDs patients, involved with an *ARID2* variant (c.2901delC, p.Asn967LysfsX2), an *ARID1B* variant (c.6532_6533insT, p.Trp2178LeufsX34), and a *SMARCC2* variant (c.2920C>G, p.Pro974Ala), which were not reported in affected individuals, established three-dimensional protein models of these variant to assess their potential pathogenic effects, and reviewed known variants in *ARID2* and *SMARCC2* among individuals with CSS/NDDs. Our findings broadened the genetic spectrum of SWI/SNF genes in NDDs and enriched phenotype profile of CSS6. We summarized the characteristic of *ARID2*, *ARID1B*, and *SMARCC2* variants to facilitate the genetic counseling and molecular diagnostics for NDDs, and our review showed that germline truncated variants occupy the overwhelming majority of *ARID2* and *ARID1B* variants and caused CSS/NDDs.

## Data Availability

The datasets presented in this study can be found in online repositories. The names of the repository/repositories and accession number(s) can be found in the article/Supplementary Material.
